# Site-Specific Phosphorylation of Histone H1.4 Is Associated with Transcription Activation

**DOI:** 10.3390/ijms21228861

**Published:** 2020-11-23

**Authors:** Ankita Saha, Christopher H. Seward, Lisa Stubbs, Craig A. Mizzen

**Affiliations:** 1 Department of Cell and Developmental Biology, School of Molecular and Cellular Biology, University of Illinois at Urbana Champaign, B107 Chemistry and Life Science Building, MC-123, 601 S. Goodwin Ave., Urbana, IL 61801, USA; seward2@illinois.edu (C.H.S.); ljstubbs@illinois.edu (L.S.); cmizzen@illinois.edu (C.A.M.); 2 Carl R. Woese Institute for Genomic Biology, University of Illinois at Urbana Champaign, Urbana, IL 61801, USA

**Keywords:** linker histone, histone1 variants, transcription, phosphorylation, transcription regulation, chromatin regulation

## Abstract

Core histone variants, such as H2A.X and H3.3, serve specialized roles in chromatin processes that depend on the genomic distributions and amino acid sequence differences of the variant proteins. Modifications of these variants alter interactions with other chromatin components and thus the protein’s functions. These inferences add to the growing arsenal of evidence against the older generic view of those linker histones as redundant repressors. Furthermore, certain modifications of specific H1 variants can confer distinct roles. On the one hand, it has been reported that the phosphorylation of H1 results in its release from chromatin and the subsequent transcription of HIV-1 genes. On the other hand, recent evidence indicates that phosphorylated H1 may in fact be associated with active promoters. This conflict suggests that different H1 isoforms and modified versions of these variants are not redundant when together but may play distinct functional roles. Here, we provide the first genome-wide evidence that when phosphorylated, the H1.4 variant remains associated with active promoters and may even play a role in transcription activation. Using novel, highly specific antibodies, we generated the first genome-wide view of the H1.4 isoform phosphorylated at serine 187 (pS187-H1.4) in estradiol-inducible MCF7 cells. We observe that pS187-H1.4 is enriched primarily at the transcription start sites (TSSs) of genes activated by estradiol treatment and depleted from those that are repressed. We also show that pS187-H1.4 associates with ‘early estrogen response’ genes and stably interacts with RNAPII. Based on the observations presented here, we propose that phosphorylation at S187 by CDK9 represents an early event required for gene activation. This event may also be involved in the release of promoter-proximal polymerases to begin elongation by interacting directly with the polymerase or other parts of the transcription machinery. Although we focused on estrogen-responsive genes, taking into account previous evidence of H1.4′s enrichment of promoters of pluripotency genes, and its involvement with rDNA activation, we propose that H1.4 phosphorylation for gene activation may be a more global observation.

## 1. Introduction

Histone H1 and the linker histones are members of a family of lysine-rich proteins that have classically been identified as structural components, playing a role in the formation of higher order chromatin structures associated with inaccessibility to transcriptional machinery [1,2]. However, more recent studies suggest that H1 is functionally dynamic, and may have a gene-specific role in the regulation of gene expression [3,4,5]. In support of this specificity, several studies have shown that only a few genes are affected by reductions in histone H1 levels; the fact that the genes affected by H1 depletion may either be up- or down-regulated argues against a general repressive function [6].

The H1 family is evolutionarily diverse, and present in non-allelic amino acid sequence-variant forms in many metazoans. Humans express eleven H1 variants; seven variants are ‘somatic’, whereas the rest are selectively expressed in germline tissues. Five of the seven somatic variants—H1.1, H1.2, H1.3, H1.4 and H1.5—are replication-dependent and are expressed predominantly during the S-phase of the cell cycle. These variants contain a more conserved amino acid sequence as compared to the replication-independent variants, namely H1.0 and H1.X. [7]

Metazoan H1 variants share a common tripartite structure containing a central globular domain (GD) that is flanked by a shorter *N*-terminal domain (NTD) and a longer *C*-terminal domain (CTD). The GD sequence is highly conserved amongst species, but there are extensive differences in the NTD and the CTD [8,9]. Fluorescence recovery after photo-bleaching (FRAP) analyses have shown that the association of H1 variants with chromatin is highly dynamic in vivo, and that the differences in the CTD regions may be key determinants of variant binding affinities and chromatin interaction dynamics [10].

Like core histones, H1 CTDs and NTDs undergo several types of posttranslational modifications, including methylation, acetylation and phosphorylation. Based on older chromatography reports and more recent mass spectrometry data, phosphorylation is observed to be the most abundant modification, progressively increasing during the cell cycle and transiently peaking at mitosis [11,12,13]. These studies also revealed that different residues are phosphorylated during interphase and mitosis and identified the specific sites of phosphorylation within the CTD. Both CDK (cyclin dependent kinase) and non-CDK motifs were identified as being phosphorylated. However, during interphase, the serine-containing consensus CDK motifs (SPXZ- S: Serine; P: Proline; X: amino acid; Z: Lysine (*L*)/arginine (*R*)) were the predominant sites of phosphorylation. These sites are also phosphorylated during mitosis, but during that time, threonine-containing CDK sites (TPXZ) and some non-CDK motifs are also phosphorylated [11,14].

Several studies have suggested that the phosphorylation event changes how H1 interacts with the chromatin. While some studies suggest that H1 phosphorylation results in its complete depletion from the chromatin [3,15], others suggest that the interaction of H1 tails with the chromatin is simply weakened, as a result of the excessive negative charge [16]. Together these studies suggest a more nuanced role for H1 and indicate that the phosphorylation event may be associated with transcriptional activation. However, since some of these studies did not use variant-specific tools, they were unable to pinpoint the differences in phosphorylated versus non-phosphorylated H1–chromatin interactions.

Earlier studies from our lab, conducted on HeLa S3 cells, identified the predominant interphase phosphorylation sites in H1.2 and H1.4 as H1.2-S173, H1.4-S172 and H1.4-S187 [17]. Using the novel, highly specific antisera developed for H1.4 phosphorylated at S187 (pS187-H1.4), we showed that pS187-H1.4 is enriched at the sites of transcription by RNAP I and II. The nuclear and nucleolar staining patterns obtained with our antisera against pS187-H1.4 and pS173-H1.2/H15 were corroborated by a different group using their own pS173H1.2/H1.5 antibody [18]. In a more recent study, we showed that the global levels of H1 phosphorylation at H1.5 H1.5-Ser18 (pS18-H1.5), H1.2/H1.5-Ser173 (pS173-H1.2/5) and pS187-H1.4 are subjected to differential regulation, and that pS187-H1.4 was associated with the maintenance of pluripotency [19]. The accumulation of the pS187-H1.4 chromatin immunoprecipitation (ChIP) signal, and the subsequent loss of that signal during differentiation at the promoters of the pluripotency genes, provided the first evidence that pS187-H1.4 is closely associated with the chromatin and is important for transcriptional activation. CDK9 was also identified in that study as the kinase responsible for this phosphorylation event [19].

Here, we provide the first genome-wide view of pS187-H1.4 dynamics using an inducible system—specifically, estradiol-responsive MCF7 cells. Using an affinity purified version of the same pS187-H1.4 antibody, we show that pS187-H1.4 is strongly associated with the promoters of active genes. Further, we show that these ‘active’ signals are quenched by a known CDK9 inhibitor, Flavopiridol. We combined pS187-H1.4 ChIP-sequencing data in conjunction with publicly available data for RNA Polymerase II (RNAP II), and Global Run-On Sequencing (GRO-seq) data derived via a similar system, to further analyze their correlation. We showed that pS187-H1.4 stably binds RNAPII and co-localizes with active RNAP II peaks, suggesting a functional interaction between the two proteins. In addition, these signals were corroborated by a GRO-seq expression dataset to show that the active pS187-H1.4 signals were seen at the promoters of genes undergoing transcriptional elongation.

Taken together, these data provide evidence for a more nuanced role of H1 and put forth the possibility of a new layer of regulation within the accepted model of transcriptional activation.

## 2. Results

### 2.1. Genome-Wide Distribution of pS187-H1.4 Displays Distinct Patterns of Enrichment

We previously generated a collection of unique, highly specific antisera using synthetic phospho-peptides and recombinant proteins and demonstrated their ability to recognize phosphorylation at single sites [17,19]. These sites are either exclusive to an individual human H1 variant or are shared between only two variants. In addition, we also raised ‘pan H1’ antisera using a whole recombinant H1 variant protein that does not distinguish between phosphorylated forms [17]. While the phospho-H1 antisera provide a measure of phosphorylation between specific sites, the pan-H1 antisera provide a comparison of the H1 variant amounts present, regardless of their phosphorylation status. Evidence of the specificity of pS187-H1.4, pan-H1.4, pS18-H1.5 and pan-H1.5 has been described previously [8,17].

Although these antisera are highly specific, their efficiency for high throughput studies was limited. Therefore, for this study, we purified pS187-H1.4 and pan-H1.4 antisera using a modified affinity chromatography approach to generate a highly efficient set of H1 antibodies. The purified pS187 H1.4 antibody was re-tested for specificity in accordance with ENCODE guidelines, as shown in Figures S1–S3. We chose MCF7 cells because they have been extensively studied, and therefore a large amount of high-throughput data are available on public platforms for comparison to and further analysis of our data [20,21,22].

**Figure 1:**
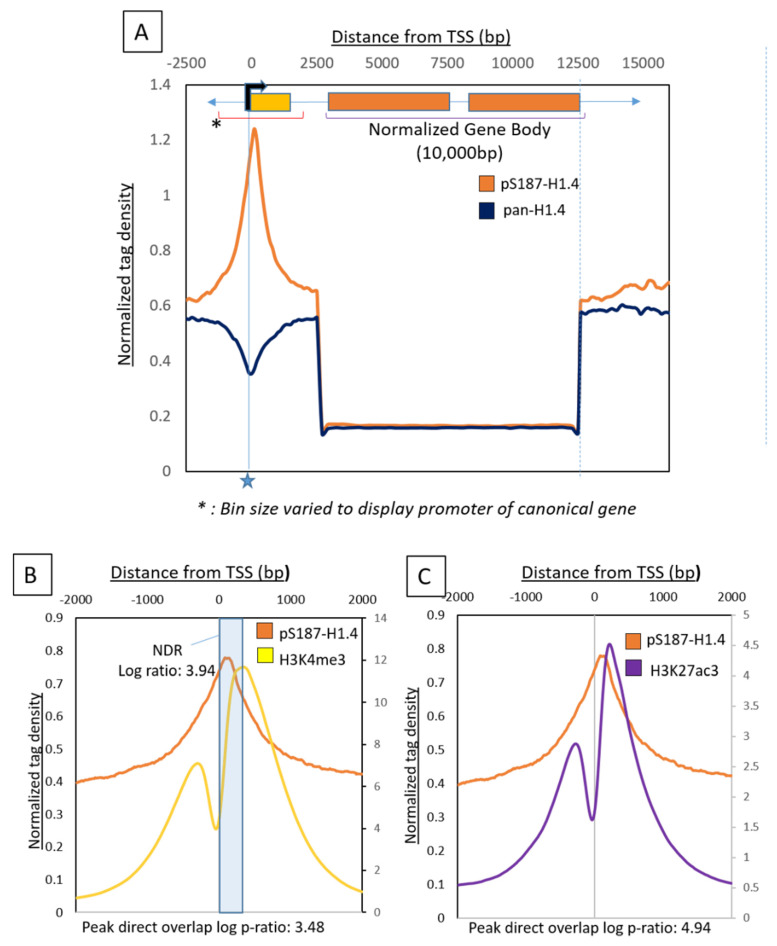
Global distribution of pS187-H1.4 and pan-H1.4 in MCF7 cells. (**A**) A metagenome profile generated with pS187-H1.4 and pan-H1.4 Chromatin Immunoprecipitation (ChIP)-sequencing data to study enrichment across a typical gene. The gene body was mathematically defined and normalized to 10 kb. The +2.5 kb and −2.5 kb regions relative to the promoter were binned at 100 bp. The 2.5 kb to 10 kb represents a normalized gene body binned at 200 bp. Typical transcription start sites (TSSs) marked by a star showed maximum enrichment of the pS187-H1.4 signal (orange trace). There was a depletion of the pS187-H1.4 and the pan-H1.4 (navy blue trace) signals along the gene body. (**B**) Aggregate plot centered on the promoter showing average pS187-H1.4 signals and its overlap with H3K4me3 signals. The signal aligned +2 Kb and −2 Kb relative to the Refseq TSSs H3K4me3, was plotted on a secondary axis (label on right side). The direct overlap of peak was calculated as a log ratio of 3.48. The H3K4me3-flanked predicted NDR region is highlighted in blue. The overlap of the pS187-H1.4 with this NDR region is 3.94. (**C**): Aggregate plot centered on the promoter showing average pS187-H1.4 signals and their overlap with H3K27ac signals. H3K27ac was plotted on a secondary axis (label on right side). The direct overlap of peak was calculated as a log ratio of 4.94.

We used the purified pS187-H1.4 and pan-H1.4 antibodies to perform chromatin immunoprecipitation (ChIP) on MCF7 cells to reveal the genome-wide distribution of all versions of the H1.4 protein (pan-H1.4) and the specifically phosphorylated version (pS187-H1.4). Peaks generated from the sequencing data were then associated with their nearest genes. We noted an enrichment of pS187-H1.4 ChIP peaks around the promoter regions (Figure 1A). The association of pS187-H1.4 peaks with promoters genome wide was significant, with a log ratio of 2.85 (Table 1) when analyzed by the HOMER software (http://homer.ucsd.edu) [23]. In striking contrast, we observed that the pan-H1.4 signal was depleted at the promoters. This pattern of pan-H1.4 localization was previously reported using another antibody [24], providing independent corroboration of our data.

In order to further characterize the status of the promoters associated with pS187-H1.4 peaks, we used H3K4me3 ChIP-Seq data from a separate study conducted on MCF7 cells [25]. The H3K4me3 epigenetic mark has widely been associated with the ‘active’ transcription state or the ‘transcription readiness’ of nearby promoters [26,27]. We plotted the average pS187-H1.4 and H3K4me3 signals around all promoters into an aggregate plot to study the trend of enrichment (Figure 1B). As illustrated by this figure, the pS187-H1.4 peaks overlap significantly with H3K4me3 peaks (Log p-ratio: 3.48). This suggests that pS187H1.4 peaks associate with ‘active’ or ‘poised’ promoters [28].

This alignment also revealed that pS187-H1.4 peaks align with the ‘dip’ between paired H3K4me3 peaks [29] (Accession number: SRX2717939), identified as H3-depleted or a ‘Nucleosome Depleted Region’ (NDR) [30]. A statistical analysis showed that the overlap between pS187H1.4 peaks and the H3K4me3-associated NDR was highly significant (log p-ratio: 3.48). In fact, it is 1.82 times more likely that a pS187-H1.4 peak will be found within this NDR than it overlapping with the H3K4me3 reads per se. Confirming that the pS187H1.4 peaks were associated with ‘active’ promoters, we also found significant overlap with another ‘active’ promoter mark, H3K27ac [25] (Figure 1C) (log ratio: 4.94) (Accession Number: SRX2717940) thereby providing multiple points of corroboration.

### 2.2. pS187-H1.4 Peaks Co-Localize with Promoter Associated RNAPII

**Figure 2:**
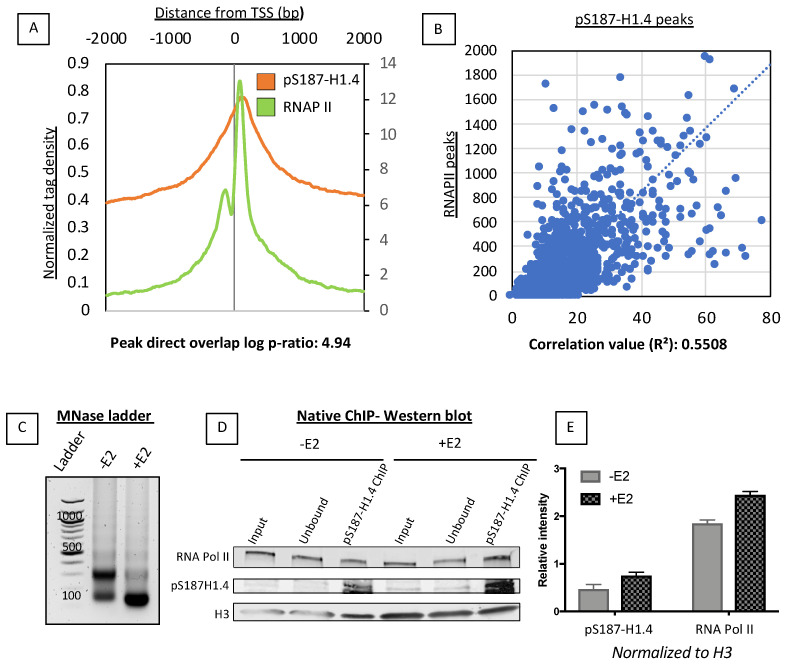
pS187-H1.4 associates with RNAPII. (**A**) Aggregate plot comparing average RNAPII (Green) and p187-H1.4 (Orange) peaks near promoter regions. The signal was aligned +2 Kb and −2 Kb relative to the Refseq TSSs. RNAPII plotted on a secondary axis (label on right side of graph). Direct overlap ratio calculated as log ratio: 4.94. (**B**) Correlation scatter plot of pS187-H1.4 and RNAPII peak overlap throughout the genome. The correlation co-efficient R^2^ calculated at 0.558. (**C**) The 6 min MNase digested DNA from estradiol untreated (−E2) and estradiol treated (+E2) MCF7 nuclei run on a 1.8% agarose gel. (**D**) Western blot following pS187-H1.4 native-ChIP. RNA pol II, pS187- H1.4 antibodies used for detection and H3 used as a loading control. (**E**) Relative intensity quantification of western blot in Figure 2D. H3 signal was used to normalize pS187-H1.4 and RNAP II signals.

Based on the promoter-enriched location of the pS187-H1.4 peaks, we then analyzed the overlap of RNA Polymerase II (RNAPII), which is usually found to be proximal to the promoters of genes that are transcriptionally ready or actively transcribed. We used MCF7 ChIP- seq data from a different study [20] (Accession Number: SRX148600) to generate aggregate plots for the RNAPII signal, and compared that signal with the average signal of pS187-H1.4 (Figure 2A). This comparison revealed a significant direct overlap between the locations of pS187-H1.4 and RNAPII proteins at the promoters in MCF7 cells (log ratio: 4.9). We also analyzed the overlap between the RNAPII and pS187-H1.4 peaks throughout the genome and confirmed a positive correlation between the two (correlation R^2^ value 0.55; Figure 2B).

In order to confirm the physical interaction of the pS187H1.4 with RNA pol II in our estradiol inducible MCF7 system, we performed native-ChIP, which does not rely on formaldehyde cross-linking, thereby providing an accurate and unbiased view of protein–protein interactions. Further, to ensure that the interaction between the RNAPII and the pS187H1.4 is within a very close range (~1–2 nucleosomes), we used micrococcal nuclease digestion to obtain a majority of mononuclesomes (Figure 2C). Following chromatin immunoprecipitation with our pS187H1.4 antibody, we analyzed the samples using a western blot. Here we see that pS187H1.4 is enriched following estradiol stimulation. Strikingly, we also see that the pS187H1.4 pulls down RNAPII, thereby demonstrating a stable physical interaction between the two, most likely within the same nucleosome. This pulldown is enriched as a result of estradiol stimulation (Figure 2D,E).

Taken together, these data show that the pS187-H1.4 peaks have a distinct pattern of distribution and strong association with ‘active’ promoters. In order to better understand the genome-wide dynamics of pS187-H1.4 at the promoters, we sought to further explore the pS187-H1.4 binding in a transcription-activating estradiol-inducible system.

### 2.3. Estradiol (E2) Stimulation Resulted in pS187-H1.4 Peak Enrichment

To ask whether pS187-H1.4 binds to promoters as they are activated, we used the purified pS187-H1.4 and pan-H1.4 antibodies to perform chromatin immunoprecipitation (ChIP) on MCF7 cells treated with 20 nM 17β-Estradiol (E2) for 30 min. This treatment is sufficient to ‘activate’ the early estrogen-regulated network of genes, following 72 h of hormone starvation [31].

The estradiol response can vary in different cell lines to the point where some genes may be oppositely regulated [32]. For example, genes involved in cell proliferation—such as CDC2, CDC6, and Thymidine kinase 1—are induced by E2 in MCF7 cells, whereas in the similar E2 responsive breast cancer cell line, 231ER+, these same genes are repressed [33]. The differences are likely due to the presence of different transcription factors and/or co-regulators in each cell type. To avoid such gene expression discrepancies, we used data from independent studies but limited only to MCF7 cells [34].

We conducted ChIP-seq in both untreated and E2-treated MCF7 cells with pS187-H1.4 and pan-H1.4 antisera, and processed the data as described in the methods section. We then used the peaks from pan-H1.4 and pS187-H1.4 before and after E2 treatment to generate metagenome profiles to visualize their distribution across the genome with respect to a normalized gene (Figure S6A). We aligned the 2013 pS187-H1.4 peaks from untreated cells and compared their distribution with 2830 peaks after E2 induction. These data showed that the E2-induced pS187-H1.4 peaks show increased levels of promoter enrichment compared to the pS187-H1.4 peaks in untreated controls. The pattern of pS187-H1.4 distribution was otherwise similar before and after hormonal induction. Furthermore, the pattern of pan-H1.4 depletion at promoters and across gene bodies remained constant before and after E2 treatment. There was only a small overall increase in pS187H1.4 signal after E2 induction (as represented in a metagenome profile) when visualized in the context of all occupied genes, as expected, since only ~15% of all genes are estradiol-responsive [20,21,35,36]. Nevertheless, an increase in overall pS187-H1.4 promoter occupation was observed.

**Figure 3:**
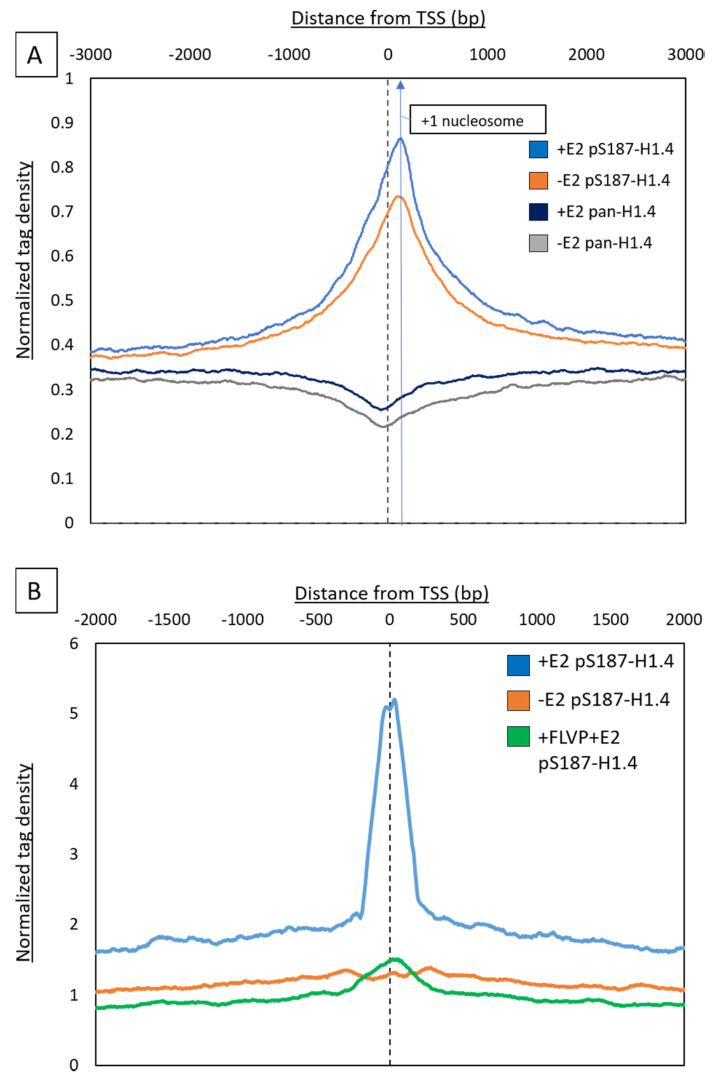
Estradiol-induced pS187-H1.4 peaks near promoter. (**A**) Aggregate plot with average pS187H1.4 peaks before (Orange) and after (Blue) estradiol (E2) treatment. The signal was aligned +3 Kb and −3 Kb relative to the Refseq TSSs. Following estradiol treatment, pS187-H1.4 is enriched. The highest signal of pS187-H1.4 shifted towards the predicted location of +1nucleosome. Pan-H1.4 before (Grey) and after (Navy blue); estradiol treatment is also seen. Depletion of signal at promoters is seen. (**B**) Aggregate plot of average 2× differential pS187-H1.4 peaks near the promoter region before (Orange) and after (Blue) estradiol treatment. Following estradiol treatment, a distinct enrichment of pS187-H1.4 over the untreated is observed. The signal was aligned +2 Kb and −2 Kb relative to the Refseq TSSs. CDK9 inhibitor treatment—Flavopiridol quenches the pS187-H1.4 signal (Green).

We focused our attention on promoter regions specifically in further analyses. First, we plotted the average pS187-H1.4 signal before and after E2 treatment, focused on the 3000 bp regions upstream and downstream of genome-wide TSSs (Figure 3A). This plot illustrates clearly that pS187H1.4 is most highly enriched at a position located approximately 150 bp from the transcription start sites, i.e., within the +1 nucleosome, where previous reports have found ‘paused’ polymerases to be bound [37,38]. We also generated aggregate plots of pS187-H1.4 peaks in E2-treated cells to test their overlap with H3K4me3, RNAPII and H3K27ac, and the enrichment of pS187-H1.4 peaks at ‘active’ promoters (Figure S6B–D). The overlap ratios were significant, with log ratios of 3.25, 4.99 and 3.14, respectively, for each ‘active’ mark. Taken together, these data show for the first time that pS187-H1.4 is associated with ‘active’ promoters and is suggestive of an interaction with transcriptional machinery. In order to further understand this enrichment pattern as a result of hormone induction, we next focused our study on the pS187-H1.4 peaks with at least a twofold increase following E2 induction.

### 2.4. X Differential pS187H1.4 Signals Enriched at Promoters and Can Be Quenched by CDK9 Inhibition

In comparisons between ChIP in E2-treated and untreated MCF7 cells, we found 727 pS187-H1.4 differential peaks with at least a twofold increase in normalized mapped reads. We visualized this increase using an aggregate plot averaging the pS187-H1.4 signal across all differential peaks, to reveal a strong preferential enrichment at the promoter (Figure 3B, blue trace). The twofold increase in pS187-H1.4 enrichment as a result of E2 treatment over the untreated control was pronounced, whereas the pan-H1.4 signal was depleted at the promoters, similar to the pattern described above.

Next, we tested the connection between CDK9 and H1.4 phosphorylation in this context, by ChIP-seq conducted after a 1 h treatment with a known CDK inhibitor, Flavopiridol (FLVP), which when used at 10 nM and for a short treatment, in this case, 1 h, is specific to CDK9 [19,39,40]. After this treatment, the pS187-H1.4 promoter signal was significantly quenched (Figure 3B—green trace). These data are consistent with a previous report by our lab, in which pS187-H1.4 was identified as a bonafide substrate for the kinase activity of CDK9 [19], and further verifies the pS187-H1.4 signal.

While the ‘metagenome’ profile and aggregate plots detect patterns of enrichment across the genome, they average the architecture of protein binding across all genes, and thus may obscure critical differences at the level of individual, or subsets of individual, loci. We used the GREAT platform (http://great.stanford.edu) to reveal the correlation of the nearest genes 1 Kb upstream and downstream of the E2-induced pS187-H1.4 peaks for previously established functional annotations. The top ten most enriched annotations from the MSigDB perturbation category are listed with their enrichment *p*-values in Table 2. This ontology category contains gene sets that represent the gene expression signatures of genetic and chemical perturbations [41]. As highlighted in the table, the strongest association of pS187-H1.4 peak-associated genes is with gene sets that were up-regulated as a result of estradiol addition in MCF7 cells, other breast cancer cell lines and even some breast tumor samples under a variety of conditions and with varying longer times of E2 treatment. This confirms that pS187-H1.4 strongly associates with promoters of bonafide E2 up-regulated genes.

**Figure 4:**
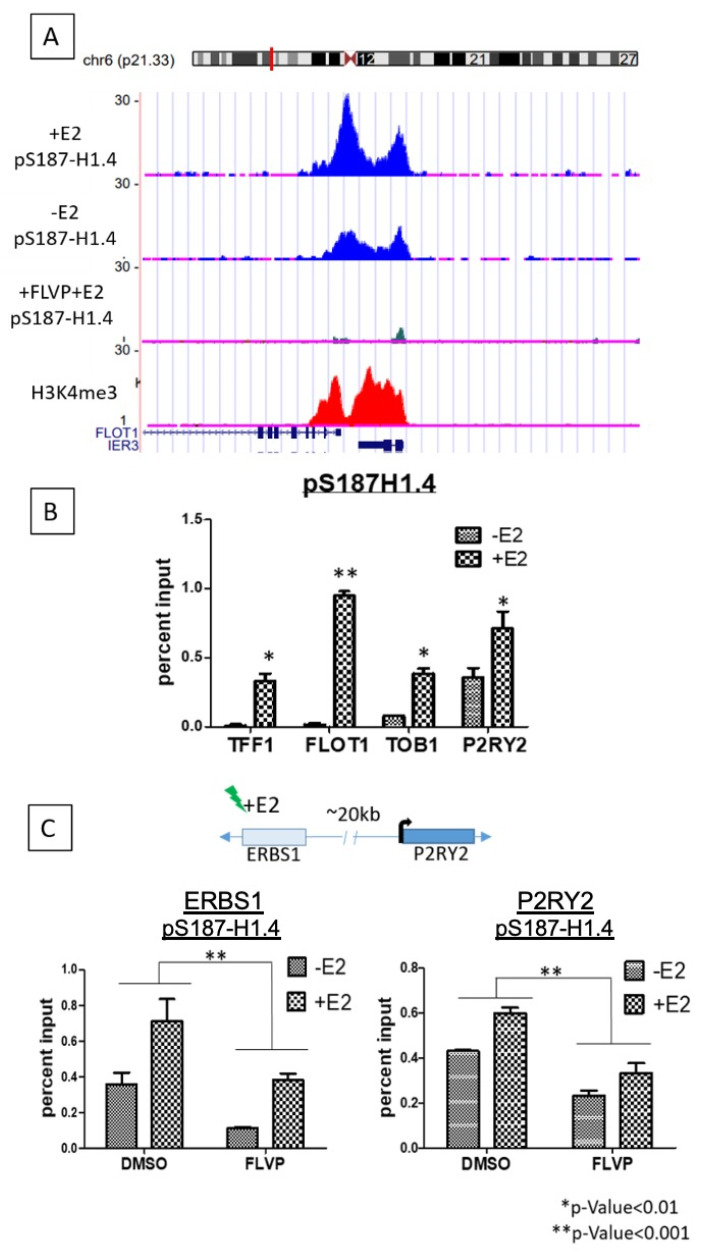
pS187-H1.4 2X differential signal verified on an individual gene level. (**A**) UCSC genome browser shot showing pS187-H1.4 signal before and after (±) the estradiol treatment of the *FLOT1* and *IER3* genes. The blue traces show pS187-H1.4 signals before and after estradiol treatment. The green track shows the pS187-H1.4 signal quenched as a result of FLVP treatment. The H3K4me3 track in red is included to show ‘active’ state of genes. (**B**) ChIP-qPCR conducted with pS187-H1.4 antibody to verify changes on an individual gene level. Changes in pS187-H1.4 enrichment as a result of E2 addition shown at the *FLOT1, TOB1, TFF1* and *P2RY2* promoters. All four genes show a significant increase in pS187-H1.4 enrichment as a result of E2 addition. (* *p*-value < 0.01, ** *p*-value < 0.001). (**C**) ChIP-qPCR conducted with pS187-H1.4 antibody to show pS187-H1.4 enrichment at the *P2RY2* gene promoter and its estrogen receptor binding site (ERBS1) that is ~20 kb upstream of the promoter. The consequent loss of enrichment as a result of FLVP treatment is also shown. Rabbit immunoglobulin (rIg) used as a control for non-specific binding. Vehicle-treated (DMSO) samples show an increase in pS187-H1.4 signal as a result of E2 addition. FLVP-treated samples show an impaired ability of pS187-H1.4 enrichment (* *p*-value < 0.01, ** *p*-value < 0.001).

To visualize this estradiol-induced pS187H1.4 enrichment in an individual gene, we used the UCSC genome browser [42] (Figure 4A). We selected the Flotilin 1 (*FLOT1*) gene from the list of genes associated with the 2X pS187-H.14 differential peaks. Here, we see that the *FLOT1* promoter is clustered very near the promoter of neighboring gene, *IER3*. Both promoters show a basal level of pS187-H1.4 signal in untreated cells; after E2 stimulation, pS187-H1.4 ChIP signals were markedly increased. In addition to this increase in pS187-H1.4 signal after estradiol treatment, we also observed a clear overlap of the pS187-H1.4 signal with that of H3K4me3, as was seen genome-wide (Figure 1B). This pS187-H1.4 signal was quenched as a result of pre-treatment FLVP, as expected. Browser views of an additional representative gene, *TOB1*, relative to housekeeping gene *ACTG1*, also demonstrated the increase in pS187-H1.4 signal as a result of estradiol treatment, the quenched pS187-H1.4 signal as a result of FLVP treatment, and the co-localization of the active signal with RNAPII (Figure S7).

To confirm the genome-wide data, we performed ChIP-qPCR to verify and quantitate the E2-induced pS187-H1.4 increase on an individual gene level, focusing on *FLOT1, TOB1*, *TFF1* and *P2RY2* (Figure 4B). We included *P2RY2* and its known estrogen receptor binding site, located approximately 20 kb upstream of the promoter and ERBS1; based on previously published GRO-sequencing data, these genes undergo transcription upregulation in response to E2 treatment [20,21]. Primers overlapping the promoter regions of these genes were designed to test ChIP enrichment by quantitative PCR (qPCR). The promoters of *FLOT1, TOB1, TFF1* and *P2RY2* all showed a significant increase in the pS187-H1.4 ChIP signal at their promoters after E2 treatment (Figure 4B). To confirm and quantitate the loss of pS187-H1.4 signal as a result of FLVP treatment, *P2RY2* and *ERBS1* were used as a representative pair. The quenching of the signal observed as a result of CDK9 inhibition supports the idea that the signal observed was in fact that of pS187-H1.4 (Figure 4C). In contrast, the ChIP signal at housekeeping genes *ACTB* and *ACTG* remained relatively unchanged as a response to E2 treatment (Figure S5), although it was reduced as a result of FLVP treatment. The minor changes observed might be attributed to the involvement of these genes in cytoskeletal rearrangements as a result of E2 induction, as previously described [43].

**Figure 5:**
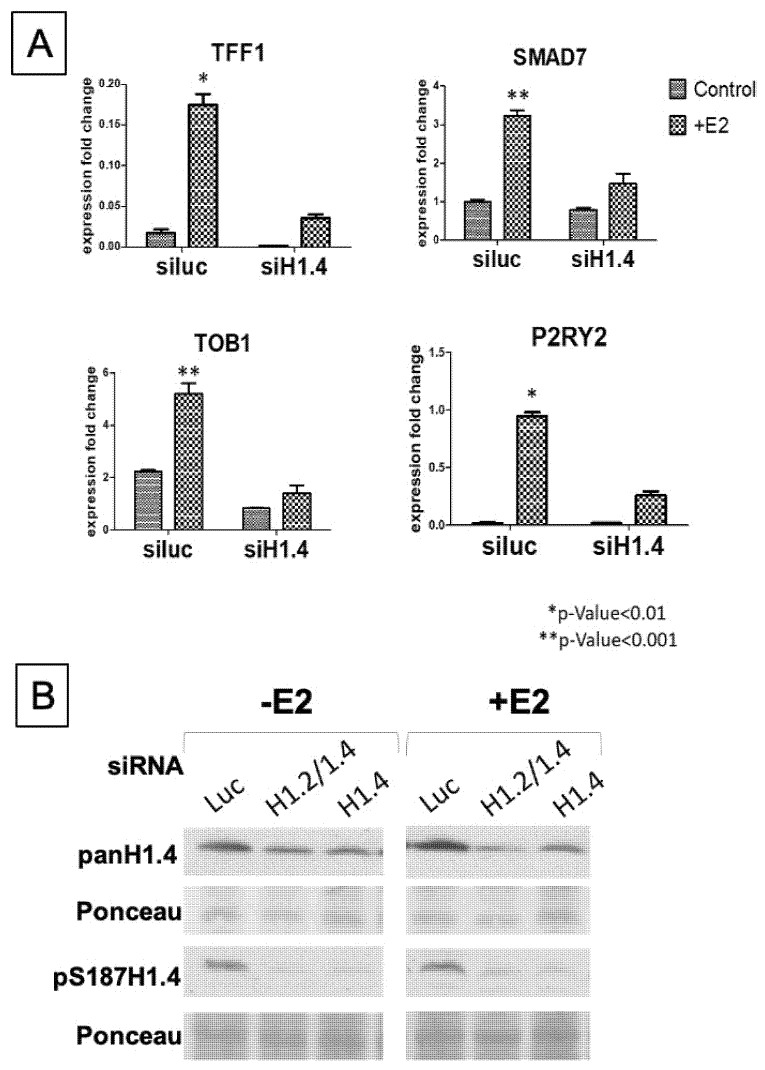
H1.4 plays a role in gene expression. (**A**) RT-qPCR to show the importance of H1.4 in gene expression. Candidate genes from our ChIP-seq study were selected to show that E2 treatment led to an increase in gene expression. This coincides with the increase in pS187-H1.4 ChIP-PCR signal described in Figure 4B. *si*Luciferase (Control) was compared to *si*H1.4 to reflect loss of gene expression in the H1.4 depleted genes. (* *p*-value < 0.01, ** *p*-value < 0.001). Signals shown as a relative fold change in expression. (**B**) Western blot comparing the levels of total H1.4 and pS187-H1.4 in response to estradiol treatment and following H1.4 knockdown. Ponceau stain showing the whole protein was used as loading control.

We also performed an siRNA-mediated knockdown assay on E2 treated and untreated cells to demonstrate the importance of H1.4. First, we observed that the pan-H1.4 protein levels are increased as a result of estradiol stimulation. Since the pan-H1.4 antibody does not discriminate between phosphorylated forms, this change is expected and speaks to the additional phosphorylated H1.4 detected. The pS187-H1.4 also shows the expected increase in signal. However, when H1.4 is knocked down, there is a reduction in pan-H1.4 signal, but a complete loss of pS187-H1.4 (Figure 5B). To ask whether this loss of pS187-H1.4 signal leads to a loss of gene expression, we performed real-time qPCR (RT-qPCR) on candidate gene transcripts derived from this study to verify their individual gene expression levels after E2 exposure in our experimental system (Figure 5A). We confirmed that the *TFF1, TOB1, TFF1* and *SMAD7* expressions were significantly induced by E2, with a twofold increase in signal, but they showed a loss of expression in response to a loss of H1.4. Putting these data in context with our observations from ChIP-PCR and ChIP-seq studies, we show that H1.4, specifically pS187-H1.4, may play an important role in gene activation.

### 2.5. pS187-H1.4 Enrichment Is Associated with Previously Identified Estradiol Responsive Genes

**Figure 6:**
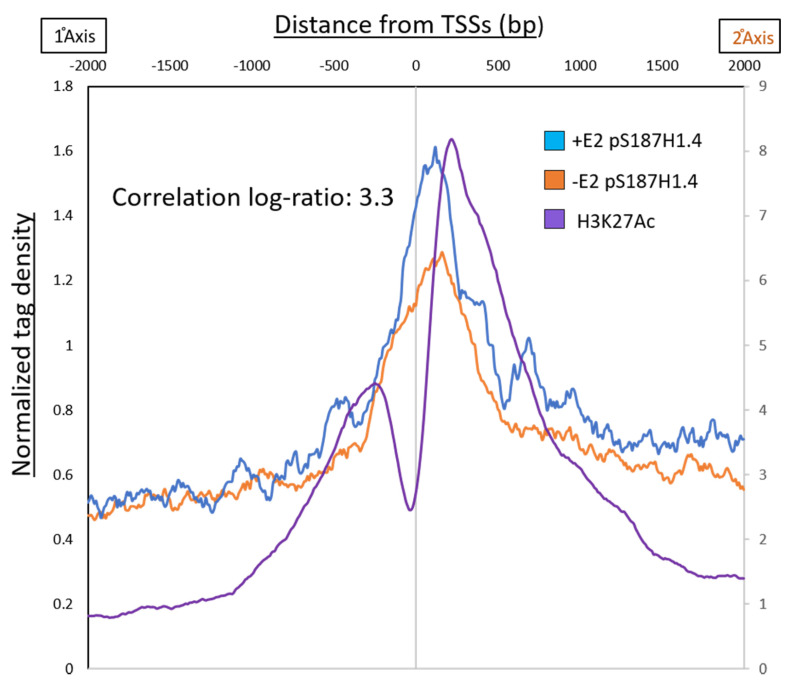
pS187-H1.4 at early responding E2-induced genes. The 196 genes previously identified as being early targets of estrogen induction were analyzed for pS187-H1.4 signal at their promoters. Aggregate plot with average pS187-H1.4 signal before (orange) and after (blue) estradiol treatment plotting, centered at refseq TSSs. A correlation ratio of pS187-H1.4 signal at these gene promoters was calculated as a log ratio (3.3).

In a study by Stendner et al., 2007, 196 genes were identified as regulated by estrogen in MCF7 cells; the responsive genes were either stimulated or repressed by E2. This study also showed that the transcription factor E2F was an early target for estrogen action and was a critical component of the hormone-induced proliferative response. To examine pS187-H1.4 enrichment at the promoters of these early E2-responsive genes, we generated an aggregate plot of the pS187-H1.4 signal around the promoters of these 196 genes (Figure 6). The pattern of pS187-H1.4 enrichment reproduced the trend that was observed in the genome-wide analysis of peaks, as observed in Figure 3A, with the pS187-H1.4 enrichment at the promoter and with the peak shifted towards the +1 nucleosome. The association of pS187H1.4 binding with verified early response elements—namely E2F binding sites—suggests that not only is the phosphorylation of H1.4 associated with active promoters, but it may be involved in the early stages of estrogen-responsive gene activation.

## 3. Material and Methods

### 3.1. Cell Culture

MCF7 cells, a kind gift from the Katzenellenbogen lab (University of Illinois at Urban Champaign) were grown in RPMI 1640 media supplemented with 5% fetal bovine serum (FBS) and 1% Penicillin-streptomycin, and were subcultured with a 0.25% trypsin-EDTA treatment followed by seeding at 1.5 × 10^6^ cells per 10 cm plate. Before use, the cells were subjected to 72 h estradiol depletion by growth in phenol-red free RPMI1640 supplemented with charcoal dextran-treated FBS (CD-FBS). Following hormone depletion, the cells were induced with 20 nM beta-estradiol (E2) dissolved in ethanol for 30 min before harvesting. In the case of drug treatments, the cells were pre-treated with Flavopiridol (NIH AIDS Reagent Program, Bethesda, MD, USA) for 1 h before E2 induction to selectively inhibit CDK9.

### 3.2. siRNA Knockdown

siRNA treatments were conducted using Lipofectamine RNAiMAX (Invitrogen, Carlsbad, CA, USA) and Luciferase control, H1.2/H1.4 and H1.4 siRNA according to manufacturer’s protocol. The cells were seeded the day prior to transfection to achieve 50–60% confluency at the time of treatment. siRNA and lipofectamine were diluted in Opti-MEM (Gibco, Gaithesberg, MD, USA), mixed, incubated for 5 min, and the complexes were then added directly to the cells now growing in phenol-red free CD-FBS Media to allow estradiol depletion along with siRNA knockdown simultaneously. After 72 h, the cells were placed in E2/ethanol for 30 min before harvesting.

### 3.3. Chromatin Immunoprecipitaiton

Chromatin immunoprecipitation was performed using protocols described before [19] with minor adjustments. Briefly, cells were cross-linked using final concentration 1% methanol-free paraformaldehyde (Thermo Fisher Scientific, Waltham, MA, USA) for 8 min quenched by 125 mM final concentration glycine for 10 min. The cells were then washed 3 times with cold PBS before scraping and resuspension in ChIP lysis buffer supplemented with protease and phosphatase inhibitors. Chromatin was then sonicated using 30 s on/off cycles for 25 min in the BiorupterTM UCD-200 (Diagenode, Liège, Belgium) sonicator to a mean sheared length of ~500 bp. Following centrifugation, the supernatants were diluted tenfold using ChIP-dilution buffer. Aliquots of 1 mL representative of 1.5–2 × 10^6^ cells were then incubated with primary antibody overnight at 4 °C. The antibody–chromatin complexes were then incubated with 50μL BSA-blocked Dynabeads (Invitrogen, Carlsbad, CA, USA) for 4 h at 4 °C and then collected using a magnetic separator. Following sequential ChIP wash-buffer and TE buffer washes, the beads were eluted using 200 μL freshly prepared elution buffer. 200 mM final concentration NaCl was added to these eluates and incubated at 65 °C overnight to reverse crosslink the immunocomplexes. Following RNAse A and Proteinase K digestion, the DNA was then purified using phenol/chloroform extraction and then precipitated using glycogen as a carrier. The precipitated DNA was then dissolved in Tris-EDTA buffer and used for qRT-PCR or Illumina sequencing.

### 3.4. ChIP-Western Blot

MCF7 cells (±E2) were collected following trypsinization. The nuclei were isolated for chromatin extraction. Briefly, the cells were washed with 0.1% tween-20 in 1XPBS, 0.1%NP40 in Tris-MgCl2 (TM2) buffer, and TM2 buffer to extract the nuclei. The nuclei were then subjected to micrococcal nuclease (2 units) treatment for 6 min before using 0.5X PBS for overnight chromatin extraction. pS187-H1.4 antibody was then added to this chromatin extract and allowed to bind overnight. Sepharose beads were used to elute the chromatin–antibody complex. This mixture was then boiled with Laemmeli buffer and then run on a 4–20% polyacrylamide gel for western blot.

### 3.5. Quantitative Real Time-PCR (qRT-PCR)

For expression analyses, RNA was extracted from cells using the trizol extraction method [44] and further purified using the Qiagen DNA purification kit. cDNA was prepared using the Superscript III first strand synthesis system (Thermo Fisher Scientific, Waltham, MA, USA) according to the manufacturer’s protocol. cDNA or ChIP products were used for qRT-PCR with SYBR-Green Mix (Applied Biosystems, Foster City, CA, USA) and primers listed in Table S1.

### 3.6. Sequencing and Bioinformatic Analysis

ChIP products were used to prepare libraries using a kit from KAPA Biosystems- Roche (Basel, Switzerland). Manufacturer protocols were followed for sheared DNA sizes 500–700 Kb. The quality control of libraries was conducted using a bioanalyzer and then sequenced using the Illumina-Hiseq 4000 by the W. M. Keck Center for Comparative and Functional Genomics at the Roy J. Carver Biotechnology Center (University of Illinois at Urbana Champaign, IL, USA). Sequence data were mapped with Bowtie2 [45] to the UCSC hg19 genome, using default settings. Mapped sequence data were analyzed for peaks using HOMER (hypergeometric optimization of motif enrichment) v4.10 [23]. Samples were converted into tag directories, and QC was performed using read mapping and GC bias statistics. Histone peaks were then called from the tag directories with default factor settings, except for the following: the local filtering was disabled (−L 0), peak size was set at 200 bp (−size 200) and minimum distance between peaks was set at 150 (-min distance 150), to increase the sensitivity of the peak calling and identify individual subunits of multi-histone peaks. After peak calling, peak files were annotated to the human hg19 genome using HOMER’s annotation script to assign peaks to the nearest genes, and associate peaks with estrogen responsive differential genes identified elsewhere by Gro-Seq [20]. BigWiggle pileup files were generated using HOMER’s makeBigWig.pl script with default settings (normalized to 10 m reads) and uploaded to a UCSC Genome Browser track hub for visualization. Differential chromatin peaks were identified using the HOMER getDifferentialPeak.pl script, looking for any peaks that changed at least twofold between conditions with a significance cutoff of 1 × 10^−4^. Genes annotated at 100 bp from differential pS187H1.4 peaks were submitted for GO analysis to DAVID and GREAT [41,46]. Metagenome profiles were generated using the HOMER makeMetaGenomeProfile.pl script using default settings. Aggregate plots were generated using the HOMER annotatePeaks.pl script with size set to 4000–6000 bp and binning set to 10 bp.

Two biological replicates of pS187-H1.4 and pan-H1.4 ChIP-sequencing were performed. The IP efficiency of replicate 1 was better than replicate 2; however, the peak enrichment patterns agreed closely, and the correlation coefficient scores calculated between the replicates were >0.94. Browser views demonstrated close to identical trends (Figure S8). Data from the first replicate were used to generate plots for better visualization. ChIP-Seq data have been deposited in the GEO database with the accession number GSE137748.

## 4. Discussion

### 4.1. pS187H1.4 Enrichment at Promoters Marks Active Genes

Previous attempts to understand the functions of histone H1 have involved mapping the distribution of all H1 variants together across the genome, under the assumption that all of the variants were interchangeable and redundant in function. However, like core histones, H1 variants also undergo distinct post-translational modifications that impart different functionalities to these proteins. Among the known modifications, phosphorylation is the most abundant [13,47], but the lack of specific antibodies has restricted the exploration of functions for these common H1 histone modifications.

In the present study, we have addressed this gap in knowledge by employing a unique antibody designed to detect a specific phosphorylated version of variant H1.4, pS187-H1.4. Early studies show that the sequence of human H1.4 shares a 93.5 sequence identity with its mouse ortholog [48,49]; this level of conservation is much higher than was observed for other variants, indicating the functional significance of H1.4. Another report also pointed to the functional significance of this variant, demonstrating that the loss of H1.4 in T47D breast cancer cells resulted in cell death [6]. H1.4 undergoes various post-translational modifications (PTMs), such as K26 methylation [50], which allows the binding of HP1 and subsequent heterochromatin function, and Gcn5-mediated H1.4K34Ac [51], which works to activate transcription by facilitating the binding of chromatin remodelers and the recruitment of transcription factors. Furthermore, ChIP and qRT-PCR studies from our laboratory of Hela, NT2 and mESC cells have implicated pS187-H1.4 in transcriptional activation, showing the enrichment of this modified variant at the promoters of active genes [17,19].

Although there have been a number of studies looking into histone PTMs, there is currently no genome-wide study describing the predominant interphase phosphorylation sites. This study provides the first genome-wide evidence of the functions of pS187-H1.4, in the context of estradiol (E2) regulation. Earlier reports have described the estrogen response as being carried out through estrogen receptor isoforms (ERα or ERβ) dimerizing with 17β-estradiol and binding to specific motifs in the DNA to elicit a transcriptional response [52,53,54]. In this well-studied system of rapid signaling, it is novel to observe that a variant of the linker histone—a family of proteins usually associated with repression—is enriched at the transcription start sites of ERα/β-E2-activated genes. The data presented here strongly support this association and raise the intriguing possibility that the H1.4, in its phosphorylated form, may add another layer of transcriptional control that has not so far been identified. For example, surprisingly, pS187H1.4 binding is enriched in the nucleosome-depleted regions that are known to harbor active binding sites for transcriptional control intermediaries, co-regulators, and even RNAPII [20,55,56]. Especially given the observation of a significant, direct overlap of pS187-H1.4 and active RNAPII across the genome, and the stable physical interaction between these two components, the possibility of a direct/indirect association with other transcriptional regulators is now open for investigation.

In a previous study by Liao and Mizzen [19], CDK9 was identified as the primary kinase responsible for the phosphorylation of S187H1.4. In this study, we have demonstrated that on a genome-wide level, the pS187-H1.4 signal at TSS is quenched upon treatment with the CDK9 inhibitor Flavopiridol (Figure 3). The CDK9 kinase is a part of the PTEF-b complex, which is responsible for the activation of RNAPII, by phosphorylating its CTD at Ser5, the negative elongation factor (NELF), leading to its depletion. This is followed by Ser2 and DRB-sensitivity inducing factor (DSIF) phosphorylation, such that it becomes a positive elongation factor and travels with the polymerase [57,58]. Despite the obvious importance of DSIF and NELF as rate-limiting factors for PolII, growing evidence suggests that there may be additional factors that may contribute to this regulation, including Gdown1 and TFIIF [59,60]. With this study, we provide evidence that not only is pS187-H1.4 a substrate of the same kinase that is involved in the regulation of PolII, but it also significantly occupies the nucleosome-depleted regions as the ‘paused’ PolII throughout the genome. Together with evidence that pS187-H1.4 physically interacts with RNAPII and also appears to accumulate at a predicted +1 nucleosome location where ‘elongating polymerases’ are known to be found [61], we speculate that phosphorylated H1.4 may be a novel factor required in the ‘activation’ of transcriptional elongation, or may even be involved in ‘priming’ the chromatin to allow transcriptional elongation by RNAPII.

### 4.2. Genome-Wide Distribution of pS187-H1.4 Reveals a Possible Interaction with Multiple Transcriptional Regulatory Pathways

The association of pS187-H1.4 with features across the genome, and particularly promoters, strengthens the hypothesis that H1.4 is important for the activation of genes via the RNAPII regulatory pathway. However, it is noteworthy to observe that a strong enrichment of pS187-H1.4 is observed in CpG Islands, which are known to mainly contain sites of active transcription (Table 1). The regulation of these chromatin regions is noteworthy in that it depends on the methylation state of the DNA and the activity and/or recruitment of polycomb factors. In special relevance to this study, CpG islands are common sites of estrogen response elements (EREs) that are responsive to estradiol [62,63]. We also observed that pS87H1.4 is strongly associated with non-coding RNAs (ncRNAs) and small nucleolar RNA (snoRNA), both of which have different regulatory mechanisms in their response to gene activation stimuli. The roles of ncRNAs in the form of lncRNAs, such as HOTAIR, MIAT and H19, in the estradiol response have only recently been described. In this context, the ncRNAs have been shown to regulate transcription by interacting with and guiding various chromatin-modifying complexes, such as Polycomb-repressive complex 2 (PRC2) and Lysine-specific demethylase 1 (LSD1) [64]. Similarly, a distinct mechanism of activation is employed by snoRNAs.

Together, this suggests that pS187H1.4 may be able to interact with multiple different transcriptional regulators, all with the final outcome of transcription activation. This suggests that, globally, pS187H1.4 may be a versatile factor with the ability to interact with various transcription regulatory components. Although this study was focused on the estradiol-based response system as a model, we observed a basal level of pS187-H1.4 proximal to the promoters. Together, these data suggest that pS187H1.4 may play a more global role in the regulation of transcription.

Since H1 has been largely regarded as a general repressor, the progress toward understanding the function and the mechanisms of action of individual H1 family members has been slow. Further, the paucity of appropriate ChIP-grade antibodies has also been a limiting factor. However, our novel, highly specific antibodies have allowed us to uncover a potential role of pS187-H1.4 in transcriptional activation for the first time, opening up a new area of study and suggesting that the roles of other specific H1 variants and their various modified forms should be further studied.

In order to further elucidate the effect of H1.4 phosphorylation on gene activation, the mechanism of action must be tested. In particular, the extent and nature of the downstream effects must be assessed in cases where the phosphorylation event is prevented. Identifying the mechanism of CDK9 recruitment to this H1.4 will also yield an insight into the nature of its activity with regard to RNAPII regulation. However, these studies raise important issues regarding the functions of H1.4 variants that suggest their broad significance in gene regulatory mechanisms, and which we are now well poised to address.

## Figures and Tables

**Table 1:** GREAT associations made with 2X differential pS187-H1.4 signal-associated genes. Top ten categories in decreasing order of *p*-value listed. Genomic Regions Enrichment of Annotations Tool (GREAT) Association MSigDB Perturbation *.

Term Name	Binom Raw *p*-Value
Genes bound by ESR1 and up-regulated by estradiol in MCF-7 cells (breast cancer) expressing constitutevly active form of AKT1	7.64 × 10^−126^
Genes bound by ESR1 and up-regulated by estradiol in MCF-7 cells (breast cancer).	3.2717 × 10^−107^
The ‘ER-alpha profile’: genes up-regulated in T47D cells upon activation of ESR1 by estradiol (E2)	1.9154 × 10^−92^
Genes upregulated in MCF7 cells (breast cancer) at 6 h of estradiol treatment.	3.2198 × 10^−65^
Genes upregulated in MCF7 cells (breast cancer) at 24 h of estradiol treatment.	1.0162 × 10^−59^
Myb-regulated genes in MCF7 (breast cancer) and lung epithelial cell lines overexpressing MYBL2, MYBL1 or MYB.	4.9913 × 10^−59^
Genes up-regulated in luminal-like breast cancer cell lines compared to the basal-like ones.	2.038 × 10^−50^
Genes whose expression negatively correlated with resistance of breast cancer cell lines to dasatinib.	2.7928 × 10^−48^
Genes regulated by ESR1 in MCF7 cells (breast cancer)	3.5364 × 10^−45^
Genes down-regulated in breast cancer tumor (formed by MCF7 xenografts) resistant to tamoxifen.	5.1164 × 10^−44^

* MSigDB Perturbation ontology contains datasets that represent gene expression signatures of genetic and chemical perturbations.

**Table 2:** Summary table of pS187-H1.4 peak correlations with genomic regions and ‘active’ genomic marks.

Log *p*-Ratio	Annotation/Peaks
**Significant association with pS187-H1.4**	
3.64	Cpg Island (h) *
2.85	Promoters (h)
2.04	Exons (h)
2.04	Protein-coding (h)
4.94	RNAPII (C) **
3.24	H3K27Ac (C)
3.48	H3K4me3 (C)
**Significant dissociation with pS187-H1.4**	
−9.92	Centromeres (h)
−0.34	Introns (h)
−1.64	Intergenic (h)

* (h): HOMER was used to identify genomic regions that were enriched in pS187H–H1.4 peaks; ** (C): ChIP–seq data from previously published studies were used to identify direct overlap with pS187–H1.4 peaks.

## Supplementary Materials

The following are available online at https://www.mdpi.com/1422-0067/21/22/8861/s1, Figure S1: Antibody validation for pS187-H1.4 and pan-H1.4 affinity purified antisera., Figure S2: Immunofluorescence of MCF7 cells (nuclei) treated with siLuc/siH1.4., Figure S3: Changes in the levels of pS187-H1.4 at promoters of genes responsive to estradiol (E2) (A–D) and housekeeping genes (E–F) as a result of siRNA treatment against H1.4., Figure S4: Immunofluorescence of MCF7 cells (nuclei) treated with DMSO/FLVP for 1 Hour., Figure S5: Changes in the levels of pS187-H1.4 at promoters of genes responsive to estradiol (E2) (A–D) and housekeeping genes (E–F) as a result of FLVP treatment against H1.4., Figure S6: Metagenome and aggregate plots demonstrating E2 induced pS187-H1.4 signal correlating with TSS and active transcription marks., Figure S7: UCSC genome browser shots of pS187-H1.4 signal (blue) before and after (±) estradiol treatment at mildly responsive housekeeping gene ACTG1 and fully responsive TOB1 genes and their co-localization with the RNAPII signals (magenta)., Figure S8: Representative browser shots of two biological repeats of pS187-H1.4 signals before and after E2 treatment as well as pre-treatment with FLVP followed by E2, at individual genes., Table S1: Primers used for ChIP-qPCR.
