# Supplementary material for: Site-Specific Phosphorylation of Histone H1.4 Is Associated with Transcription Activation
Source: Int J Mol Sci. 2020 Nov 23;21(22):8861. doi: 10.3390/ijms21228861 (PMC7700352; doi:10.3390/ijms21228861)
Supplement: Supplementary file 1 [file ijms-21-08861-s001.pdf]

Supplementary Figures

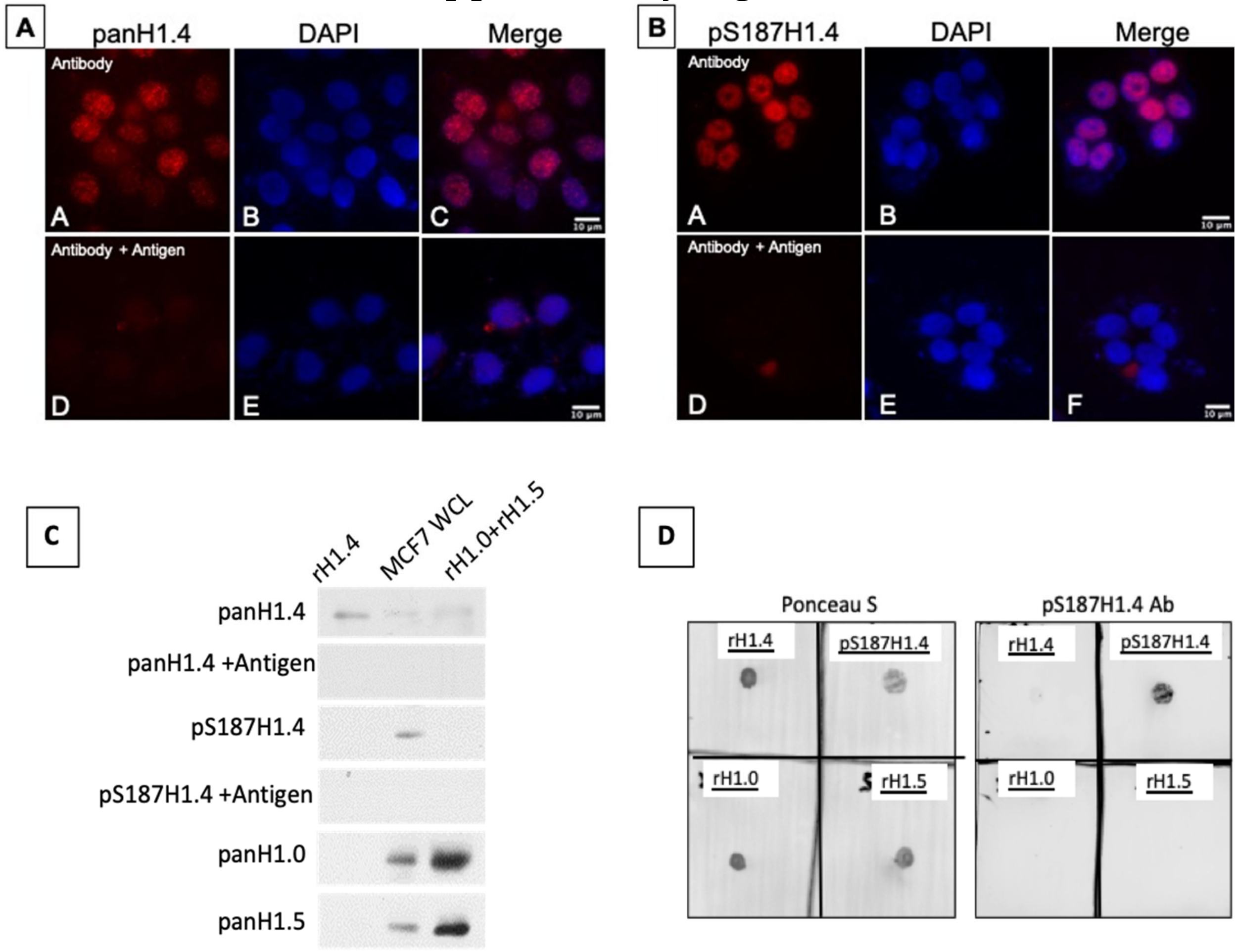

Supplementary Figure 1. Antibody validation for pS187-H1.4 and pan-H1.4 affinity purified antisera. (**A**) Immunofluorescence performed on MCF7 cells to show pan-H1.4 staining (panels **A**–**C**). The pan-H1.4 signal was quenched when the immunofluorescence was performed with antigen-adsorbed pan H1.4 antibody (Panels **D**–**F**). (**B**) Immunofluorescent staining performed on MCF7 cells to show pS187-H1.4 staining (panels **A**–**C**). The pS187-H1.4 signal was quenched when the immunofluorescence was performed with antigen-adsorbed pS187-H1.4 antibody (Panels **D**–**F)**. Scale bar of 10µm in DAPI merge panels representative of the preceding images. (**C**) Western blot performed with recombinant H1.4, MCF7 whole cell lysate (WCL) and a mixture of recombinant H1.0 and H1.5. pan-H1.4 and pS187-H1.4 antibodies used to show specific signal and demonstrate quenching when antibody was antigen adsorbed. (**D**) Dotblot performed to demonstrate specificity of pS187-H1.4 antibody. Left panel show ponceau staining of the recombinant H1.4, H1.0, H1.5 and pS187H1.4 peptide. The right panel shows specific staining with pS187-H1.4 antibody.

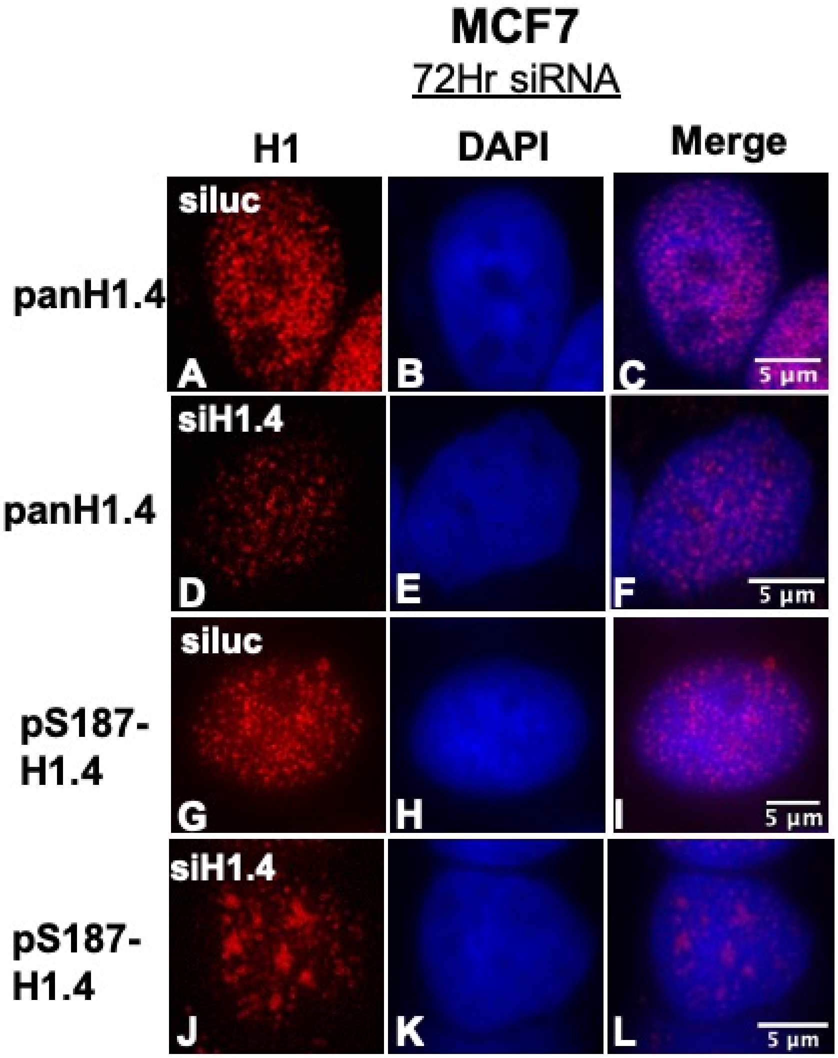

Supplementary Figure 2: Immunofluorescence of MCF7 cells (nuclei) treated with siLuc/siH1.4. Panels (**A**–**F)** shows pan-H1.4 staining of nuclei treated with siluc (**A**–**C**) and siH1.4 (**D**–**F**). Panels **G**–**L** shows pS187-H1.4 staining of nuclei treated with siluc (**G**–**I**) and siH1.4 (**J**–**L**). Scale bar of 5μm in DAPI merge panels representative of the preceding images

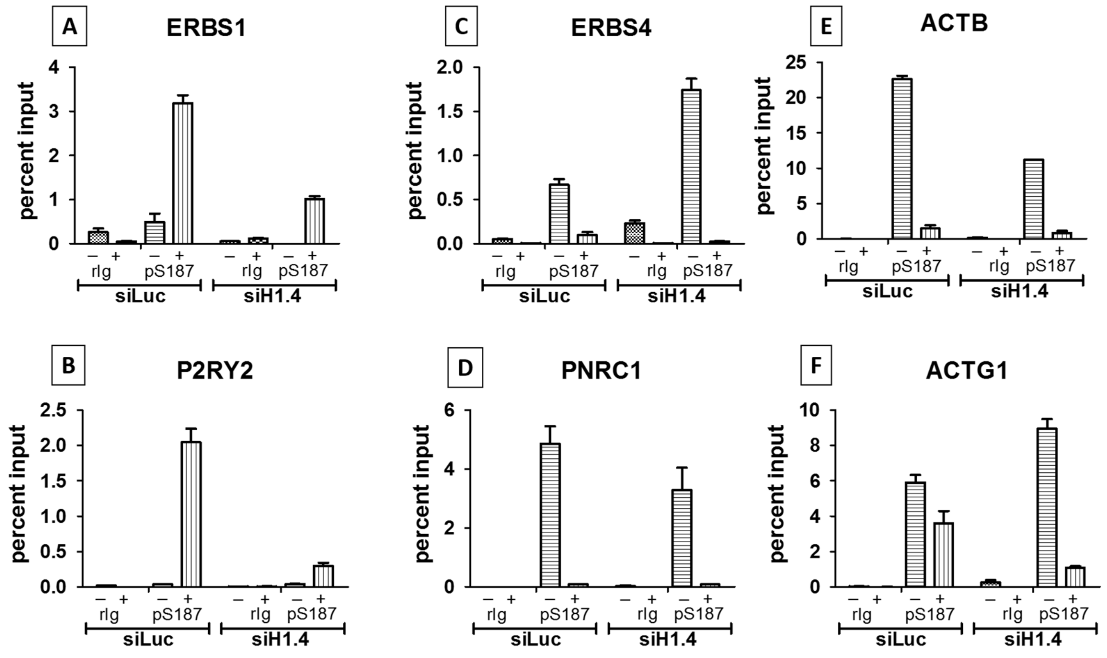

Supplementary Figure 3. Changes in the levels of pS187-H1.4 at promoters of genes responsive to estradiol (E2) (**A**–**D**) and housekeeping genes (**E**–**F**) as a result of siRNA treatment against H1.4 were assessed by ChIP-qPCR. (**A**–**B**). The levels of pS187-H1.4 at the promoters of P2RY2 and ERBS1 rise as a result of estradiol treatment. These levels drop as a result of H1.4 knockdown. (**C**–**D**) The levels of pS187-H1.4 at the promoters of PNRC1 and ERBS4 are repressed as a result of estradiol treatment. These levels diminish further as a result of siRNA mediated H1.4 knockdown. **E**–**F**) ACTB and ACTG1 appear to be repressed as a result of estradiol treatment. However, the pS187-H1.4 levels at the promoter of ACTB result in a further decrease of signal. pS187-H1.4 appears to increase as a result of the H1.4 knockdown but is reduced more than the siluc control when induced with estradiol. In all cases, negative control ChIP assays were employed non-immune rabbit immunoglobulin (rIg) in place of primary antisera.

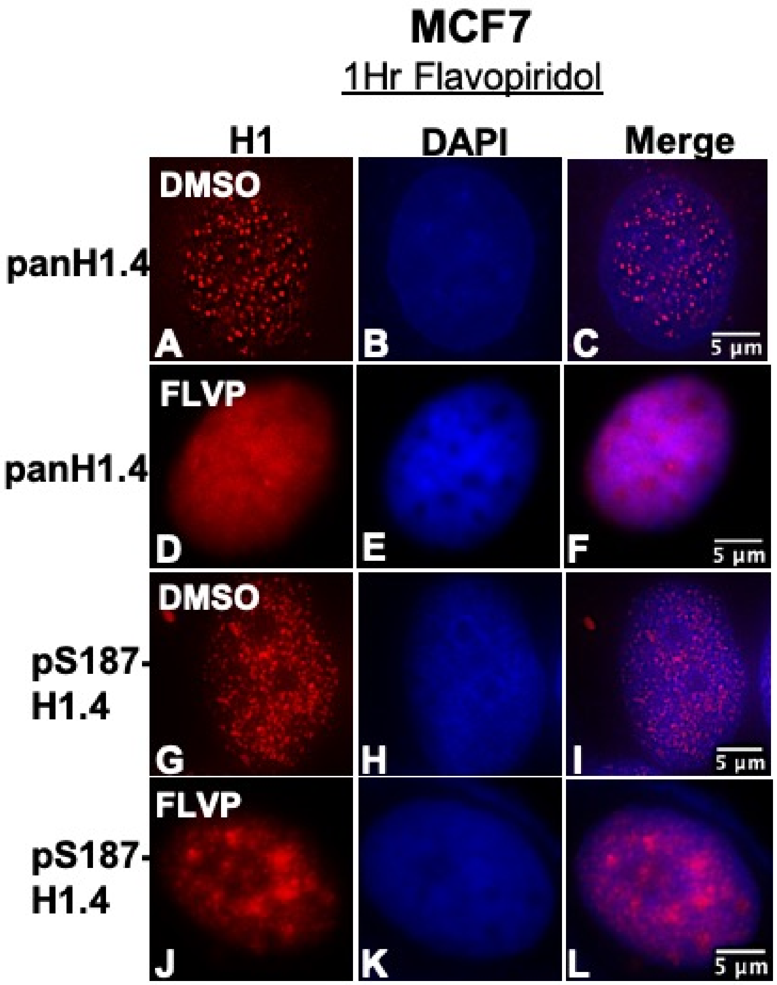

Supplementary Figure 4: Immunofluorescence of MCF7 cells (nuclei) treated with DMSO/FLVP for 1 Hour. Panels **A**–**F** shows pan-H1.4 staining of nuclei treated with DMSO (**A**–**C**) and FLVP (**D**–**F**). Panels **G**–**L** shows pS187-H1.4 staining of nuclei treated with DMSO (**G**–**I**) and FLVP (**J**–**L**).

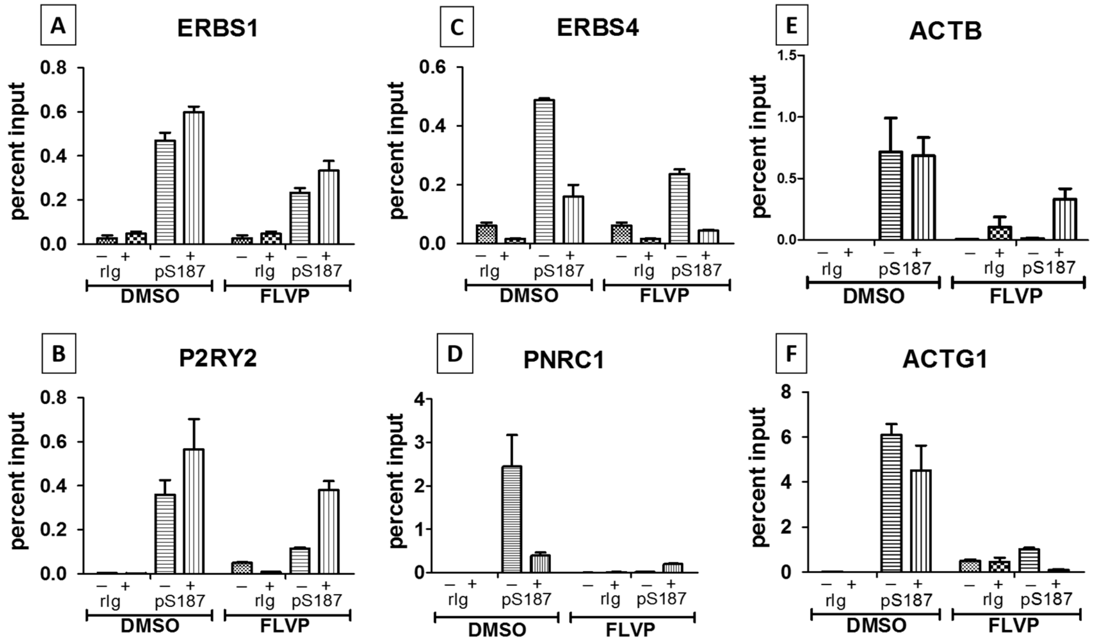

Supplementary Figure 5: Changes in the levels of pS187-H1.4 at promoters of genes responsive to estradiol (E2) (**A**–**D**) and housekeeping genes (**E**–**F**) as a result of FLVP treatment against H1.4 were assessed by ChIP-qPCR. (**A**–**B**) The levels of pS187-H1.4 at the promoters of P2RY2 and ERBS1 rise as a result of estradiol treatment. These levels drop as a result of H1.4 knockdown. **C**–**D**) The levels of pS187-H1.4 at the promoters of PNRC1 and ERBS4 are repressed as a result of estradiol treatment. These levels diminish further as a result of FLVP. **E**–**F**) ACTB and ACTG1 appear to be repressed as a result of estradiol treatment. However, the pS187-H1.4 levels at the promoter of ACTB result in a further decrease of signal. pS187-H1.4 appears to increase as a result of the H1.4 knockdown but is reduced more than the siluc control when induced with estradiol. In all cases, negative control ChIP assays were employed non-immune rabbit immunoglobulin (rIg) in place of primary antisera.

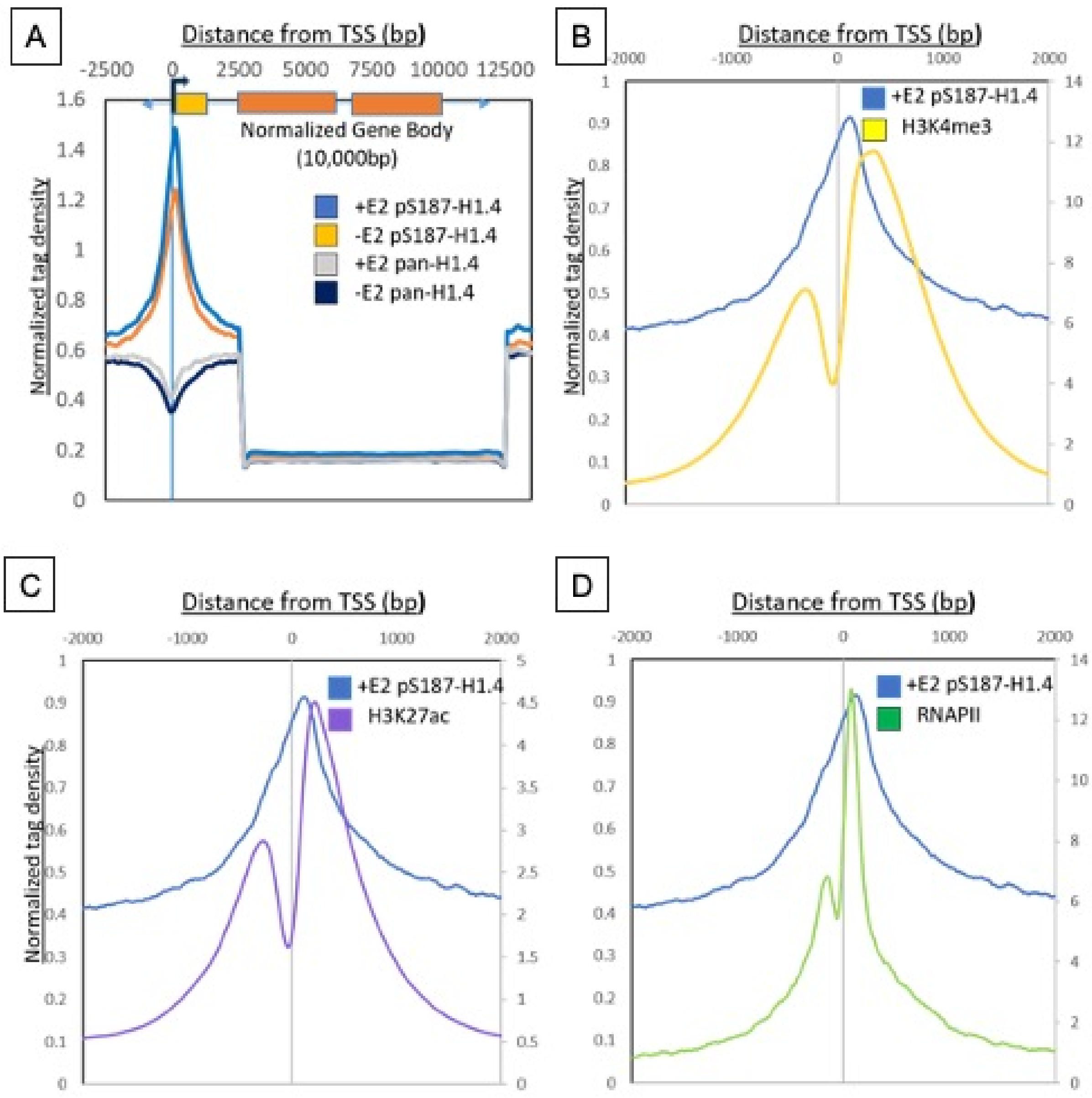

Supplementary Figure 6: Metagenome and aggregate plots demonstrating E2 induced pS187-H1.4 signal correlating with TSS and active transcription marks. (**A**) A metagenome profile generated with E2 induced pS187-H1.4 and pan-H1.4 ChIP-sequencing data to study enrichment across a typical gene. The gene body was mathematically defined and normalized to 10kb. +2.5kb and -2.5kb regions relative to the promoter were binned at 100bp. 2.5kb to 10kb represents a normalized gene body binned at 200bp. Typical transcription start sites (TSSs) showed maximum enrichment of the E2 induced pS187-H1.4 signal (blue trace). (**B**): Aggregate plot centered on the promoter showing average E2 induced pS187-H1.4 signals and its overlap with H3K4me3 signals. The signal was aligned +2Kb and -2Kb relative to the Refseq TSSs H3K4me3 was plotted on a secondary axis (label on right side). Overlap ratio: 3.25 (**C**): Aggregate plot centered on the promoter showing average pS187-H1.4 signals and its overlap with H3K27ac signals. H3K27ac was plotted on a secondary axis (label on right side). Overlap ratio: 4.99. (**D**) Aggregate plot centered on the promoter showing average pS187-H1.4 signals and its overlap with RNAPII signals. Overlap ratio: 3.14

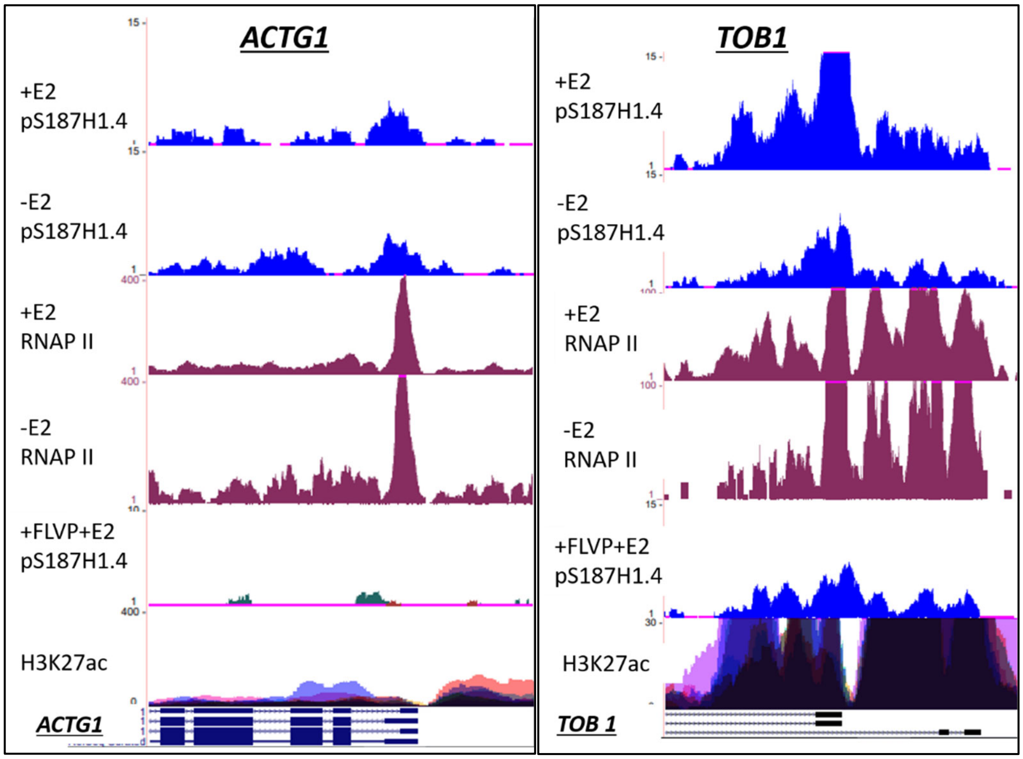

Supplementary Figure 7: UCSC genome browser shots of pS187-H1.4 signal (blue) before and after (±) estradiol treatment at mildly responsive housekeeping gene ACTG1 and fully responsive TOB1 genes and their co-localization with the RNAPII signals (magenta).

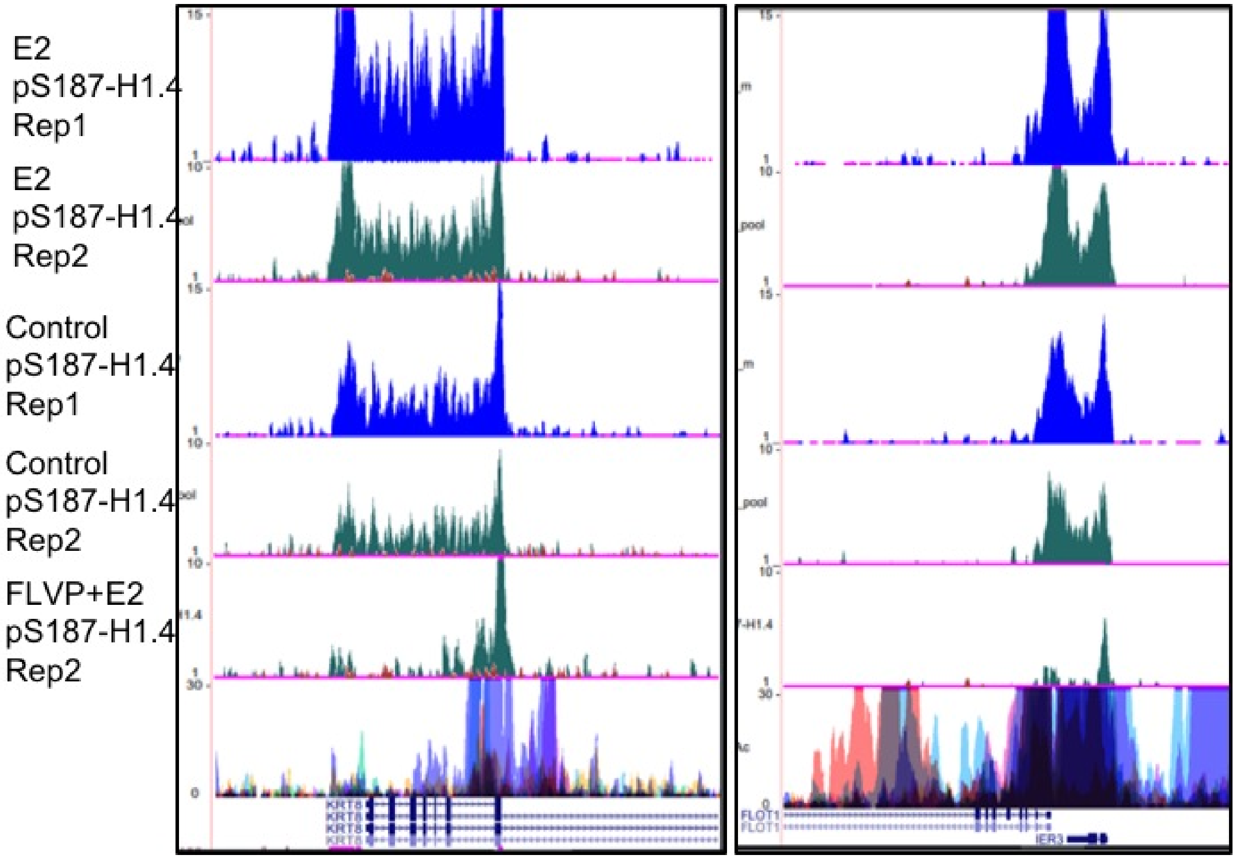

Supplementary Figure 8: Representative browser shots of two biological repeats of pS187-H1.4 signals before and after E2 treatment as well as pre-treatment with FLVP followed by E2, at individual genes. Repeat 1 is shown in blue and repeat 2 is shown in green. Trends observed are close to identical with an agreement value > 0.94.

Supplementary Table 1: Primers used for ChIP-qPCR.

Gene Name	Forward Primer	Reverse Primer
TFF1	GAACAAGGTGATCTGCGCCC	CACTGTACACGTCTCTGTCTGG
FLOT1	AAGCTGCCCCAGGTGGCAGAG G	TGTTCTCAAAGGCTTGTGATTCACC
TOB1	GCTGTGTGGAGAAGTGAGCG	CTTGGGAGATCGCCGTTAGT
SMAD7	TGGGTCCAAGGACAGATGTA	ACTCTCTGCATTGGTGAAGC
SOX2	GGGGAAAGTAGTTTGCTGCC	GCTTAAGCCTGGGGCTC
POU5F1	CTTCGCAAGCCCTCATTT	AGGTCCGAGGATCAACCC
ACTG1	CGGCTTTCGGAAAGATCG	GAGCGGCGGAAGAACAGA
ACTB	GAAAGTTGCCTTTTATGGCTCG	TTACCTGGCGGCGGGTGT
P2RY2	CGGTGGACTTAGCTCTGAGG	GCCTCCAGATGGGTCTATGA
PNRC1	TCGCTCAGCAACGAAGAGAG	TCGCTCAGCAACGAAGAGAG
ERBS1	AGGCAAATCCATTGTCATCC	AACTGGCTGGATCTTGAAGC
ERBS4	GGCATAGCTAGGACCTCACC	GAGGGAGGAAAGTGGCTTCT
